# User Perceptions of a Technology-Based Social Memory Aid for Persons With Memory Concerns

**DOI:** 10.1093/geroni/igab046.3751

**Published:** 2021-12-17

**Authors:** Brenna Horn, Elizabeth Albers, Eric Jutkowitz, Jessica Finlay, Lauren Mitchell, Ashley Millenbah, Jude Mikal

**Affiliations:** 1 University of Minnesota/ Families and Long-Term Care Projects, Minneapolis, Minnesota, United States; 2 University of Minnesota, Minneapolis, Minnesota, United States; 3 Brown University, Brown University, Rhode Island, United States; 4 University of Michigan, Ann Arbor, Michigan, United States; 5 Emmanuel College, Boston, Massachusetts, United States; 6 University of Minnesota, University of Minnesota, Minnesota, United States

## Abstract

People with memory concerns (PWMC) are likely to experience social withdrawal and isolation. Although assistive technologies and memory aids are available to support PWMC and their family caregivers, few have been shown to improve social engagement. This study aimed to gain perspectives of PWMC and their family caregivers on the feasibility and utility of a technology-based social memory aid. We recruited 20 dyads of people with mild to moderate memory concerns and family caregivers to evaluate Smartwatch Reminder (SR), a notification system that provides a name, relationship, and photograph of nearby social contacts to aid in recognition. Dyads viewed a demonstration of the SR prototype, and then participated in semi-structured interviews over Zoom video conferencing from June to August, 2020. Interview transcripts were analyzed using thematic analysis, with analyses completed in August 2021. Our findings indicate that participants anticipated important benefits from using the technology, and thought the technology would be easy to use. Participants perceived that the memory aid could benefit them now and in the future by alleviating socialization-related stress for both members of the care dyad; however, certain features of SR restricted the potential benefits, such as the requirement that social contacts have the SR application, and that prompts are only provided during social encounters. Our findings will inform future technology-enabled memory aid development to improve social engagement for PWMC and support family caregivers.

